# Prevalence of medication discrepancies in patients with cirrhosis: a pilot study

**DOI:** 10.1186/s12876-016-0530-4

**Published:** 2016-09-13

**Authors:** Kelly L. Hayward, Patricia C. Valery, W. Neil Cottrell, Katharine M. Irvine, Leigh U. Horsfall, Caroline J. Tallis, Veronique S. Chachay, Brittany J Ruffin, Jennifer H. Martin, Elizabeth E. Powell

**Affiliations:** 1 School of Medicine, The University of Queensland, Translational Research Institute, Brisbane, Australia; 2 Pharmacy Department, Princess Alexandra Hospital, Brisbane, Australia; 3 QIMR Berghofer Medical Research Institute, Brisbane, Australia; 4 School of Pharmacy, The University of Queensland, Brisbane, Australia; 5 Centre for Liver Disease Research, The University of Queensland, Brisbane, Australia; 6 Department of Gastroenterology and Hepatology, Princess Alexandra Hospital, Woolloongabba 4102, Brisbane, Queensland Australia; 7 School of Human Movement and Nutrition Sciences, The University of Queensland, Brisbane, Australia; 8 School of Medicine and Public Health, The University of Newcastle, Newcastle, Australia

**Keywords:** Medication reconciliation, Medication adherence, Liver cirrhosis, Complementary therapies, Ambulatory care

## Abstract

**Background:**

Cirrhosis patients are prescribed multiple medications for their liver disease and comorbidities. Discrepancies between medicines consumed by patients and those documented in the medical record may contribute to patient harm and impair disease management. The aim of the present study was to assess the magnitude and types of discrepancies among patient-reported and medical record-documented medications in patients with cirrhosis, and examine factors associated with such discrepancies.

**Methods:**

Fifty patients who attended a hospital hepatology outpatient clinic were interviewed using a questionnaire composed of mixed short-response and multiple-choice questions. Patients’ reported medication use was compared with documentation in the hospital medical records and pharmacy database. Medication adherence was assessed using the 8-question ©Morisky Medication Adherence Scale (MMAS-8). The multivariate logistic regression model was constructed using clinically relevant and/or statistically significant variables as determined by univariate analysis. All p-values were 2-sided (α = 0.05).

**Results:**

Twenty-seven patients (54.0 %) had ≥1 discrepancy between reported and documented medicines. Patients with ≥1 discrepancy were older (*p* = 0.04) and multivariate analysis identified taking ≥5 conventional medicines or having a ‘low’ or ‘medium’ adherence ranking as independent predictors of discrepancy (adjusted OR 11.0 (95 % CI 1.8–67.4), 20.7 (95 % CI 1.3–337.7) and 49.0 (95 % CI 3.3–718.5) respectively). Concordance was highest for liver disease medicines (71.9 %) and lowest for complementary and alternative medicines (14.5 %) and respiratory medicines (0 %).

**Conclusion:**

There is significant discrepancy between sources of patient medication information within the hepatology clinic. Medication reconciliation and medicines-management intervention may address the complex relationship between medication discrepancies, number of medications and patient adherence identified in this study.

## Background

Liver disease is gaining global recognition as an important chronic health disorder, due to increasing prevalence of non-alcoholic fatty liver disease (NAFLD), hazardous alcohol intake and viral hepatitis [1]. Regardless of aetiology, morbidity and mortality occurs predominantly among patients with cirrhosis, a late stage of progressive fibrosis with liver vascular and architectural alterations. Clinically, cirrhosis is defined as “compensated”, a latency period with median survival times of more than 12 years, or “decompensated”, a rapidly progressive phase marked by complications of portal hypertension or liver insufficiency and median survival times of less than 2 years [2].

The morbidity and health care costs associated with the complications of decompensated cirrhosis are substantial, as people require complex medical care and have very high use of hospital services [3, 4]. With the growing prevalence of liver cirrhosis worldwide, it is becoming increasingly important to identify potentially-modifiable factors that may contribute to disease burden.

People with cirrhosis are often prescribed multiple medications for therapeutic or prophylactic use [3] and the number of medications prescribed on hospital discharge is a risk factor for early readmission [5]. Although the precise reason for this has not been established, and increased medication use is common in people with more severe illness, medication misuse and non-adherence may have contributed. Polypharmacy is strongly related to poor adherence and both factors have also been associated with medication misuse and a higher prevalence of discrepancies between patient-reported and clinician-documented medications [6, 7]. Discrepancies between the type and frequency of medications prescribed by clinicians and the drugs actually consumed by patients may contribute to patient harm or reduce the efficacy of therapy. Unresolved medication discrepancies have been correlated with increased length of hospital stay, readmission within 30 days and adverse events post-discharge [8–10].

In contrast to other chronic diseases, the prevalence of medication discrepancies has not been examined in patients with cirrhosis. Examination of the types and magnitude of discrepancies that are present and the potential harms associated with them is important to improve clinician recognition of this potential barrier to care, especially with the growing push for treatment and follow-up of chronic liver disease (CLD) patients in community settings.

## Aims

To assess the magnitude and types of discrepancies between reported and documented medications in patients with cirrhosis seen in a hospital hepatology clinic, and examine factors associated with such discrepancies.

## Methods

### Patients and clinical data

A convenience sample of 50 English-speaking patients with cirrhosis were invited to participate when they attended the hepatology outpatient clinic at the Princess Alexandra Hospital (Brisbane, Australia) from August to December 2014.

Participants (and carers/family members if present) were interviewed by the research co-ordinator using a questionnaire composed of mixed short-response and multiple-choice questions designed to elicit demographic information, patient knowledge of their medications and liver disease and related lifestyle factors. Self-reported adherence to cirrhosis medications was evaluated using the eight-item Morisky Medication Adherence Scale© (MMAS-8) with approval from the developer [11–13]. The MMAS-8 is a previously validated questionnaire used to estimate self-reported adherence to treatment and is widely used in chronic diseases. It consists of seven questions with “yes” or “no” alternatives, and one item featuring a 5-point Likert scale. The MMAS-8 scores range from 0–8, with levels of adherence classified as: high adherence (score 8); medium adherence (score 6–7.75); and low adherence (score <6).

Patients’ medical records, standard biochemical and serological assays and liver imaging were used to confirm the diagnosis of liver disease and cirrhosis. In addition, Fibroscan®, gastroscopy and histological assessment of a liver biopsy were also used, if performed. The severity of liver disease was evaluated using the Child-Turcotte-Pugh classification.

### Patient reported medications

Subjects were asked to list the dose, frequency and indication for each of their medicines and specifically prompted for over-the-counter (OTC) and complementary medicines (CAMs). Qualitative questions were also asked throughout the interview to elicit individual medication-taking behaviour. Medications were not actively verified with other sources such as the GP or local pharmacy, as medication reconciliation was not standard practice within the clinic at the time this study was conducted.

### Documented medications

Medications were considered current if documented less than 3 months prior to patient interview, without subsequent documentation of cessation or modification. Each patient’s medical record and the pharmacy database ‘ELMs’ (Enterprise-wide Liaison Medication System) were interrogated to determine documented medications and compare to patient responses. ELMs is a state-wide hospital pharmacy database that is routinely updated by hospital pharmacists at the point of admission and/or discharge from hospital. Within the outpatient hepatology clinic there was no assigned clinician or assistant who routinely verified and updated the patient’s medication list. Consequently, medications were not consistently recorded within the outpatient section of the medical record, and thus correspondence letters from GPs, other specialists and admission notes were also used to determine documented medications.

### Data analysis

‘Medication discrepancy’ was defined as a difference between what was reported by the patient and what was documented in the medical record or in ELMs. Documented medications in the ELMs database which were annotated or classified as ‘temporary’ by the study clinicians (antibiotic courses, post-operative analgesia, some PRN medications, medicines with a documented cessation date) were not included in the discrepancy analysis.

Correlation between reported and documented medicine name, dose, frequency and indication was attempted, but due to patient ambiguity and limited chart documentation of dosage, only the name of medications could be analysed for this study. The clinical significance of discrepancies was determined by a panel of clinicians experienced in treating cirrhosis patients (pharmacist, hepatologist and nurse). A significant discrepancy was defined as one which may lead to potential harm within 7 days if the patient was administered, or not administered, a drug due to misdocumentation or misreporting. Medications which were in agreement between 2 sources were considered ‘concordant’.

Medications were categorised as ‘conventional medications’ (including prescription medicines, OTCs, vitamins and protein supplements prescribed for the treatment of cirrhosis-related complications and other comorbidities) or ‘CAMs’. Medications were grouped into 12 drug-disease categories: liver, gastrointestinal-luminal, cardiovascular, diabetes, psychomodulators, analgesia, CAMs, respiratory and ‘others’. Proton-pump inhibitors (PPIs) were classified as a ‘liver’ medication in patients with gastric and/or oesophageal varices or a ‘gastro-luminal’ medication when prescribed for gastro-oesophageal reflux disease. Liver disease medications were further analysed by drug name and/or indication.

### Statistical analysis

Data analysis was conducted using SPSS Inc. version 20.0 (College Station TX: StatCorp LP; 2013). Participant characteristics are presented as means and standard deviation (normally distributed data), and proportions. Univariate analysis was performed using Pearson’s Chi-squared analysis or Fisher’s Exact test for categorical data (proportions), and t-test for normally-distributed data (means). The association between medication discrepancy and demographic and clinical variables was determined by calculating the odds ratios (OR) and 95 % confidence interval (CI). The multivariate logistic regression analysis model was constructed by testing variables of clinical relevance and/or statistical significance as determined by univariate analysis. The Hosmer-Lemeshow test was performed on selected models to assess goodness-of-fit. The final model was used to assess associations after adjustment for the total number of conventional medications taken by patients (excluding CAMs), and MMAS ranking. Interactions between individual variables (age, gender, regular general practitioner (GP), comorbidities, number of conventional medications and MMAS ranking) were not found to be statistically significant. All *p* values were 2-sided and statistical significance was set at alpha = 0.05.

## Results

### Patient characteristics

Fifty-three cirrhotic patients who attended the hepatology clinic at the Princess Alexandra Hospital were invited to participate; 50 (94.0 %) were interviewed, and three declined to participate. Overall, the mean age of participants was 58.5 (±10.2) years; 39 patients (78.0 %) were men and 43 (86.0 %) were Caucasian. Primary liver disease aetiology was Hepatitis C in 26 patients; non-alcoholic steatohepatitis in 11; alcoholic liver disease in 10; Hepatitis B in one; primary biliary cirrhosis in one; cryptogenic in one. Twenty patients (40.0 %) had decompensated cirrhosis at the time of interview, including five with a history of hepatic encephalopathy, 14 with ascites and 15 with oesophageal/gastric varices.

A total of 307 medications were identified from all sources; 244 were classified as ‘conventional’, 63 as CAMs, and the drug-disease classes comprised liver-related (22.8 %), CAM (20.5 %), cardiovascular (15.6 %), diabetes (8.8 %), “other” (8.5 %), psychomodulators (8.1 %), analgesia (7.2 %), gastro-luminal (4.9 %) and respiratory (3.6 %) medications. Seven patients (14.0 %) stated that they took no medications, however two disclosed OTC/CAMs when prompted and one had salbutamol ‘when required’ documented within their medical record. Twenty-seven patients (54.0 %) had ≥5 conventional medications identified from all sources.

### Medication discrepancies

**Fig. 1:**
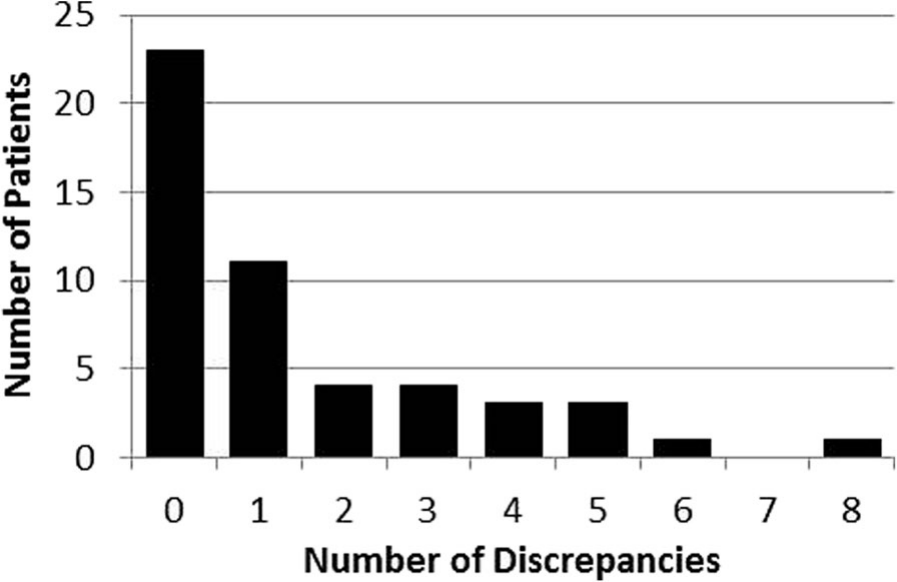
Number of discrepancies between patient-reported conventional medications (including prescribed CAMs) and their medical record

Significant discrepancies between patient-reported conventional medications (including prescribed CAMs) and the medical record were present in 27 patients (54.0 %). All 27 patients reported conventional medications which were not recorded in the medical record and 16 patients also did not report conventional medications that were documented in the medical record. Twenty-four percent of patients had three or more discrepancies among conventional medicines identified (Fig. 1).

Sixteen patients had medications recorded in the ELMs database. Of these 16 patients, discrepancies in conventional medicines were present in 11 patients (68.8 %); five reported conventional medications which were not recorded in ELMs, and nine had medications recorded in ELMs which they did not report.

**Fig. 2:**
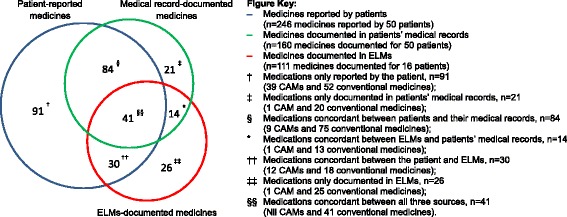
Venn distribution of medications reported by patients, documented in their medical records and recorded in ELMs. Overlap represents medications that were concordant between sources. Total number of medications = 307 (*n* = 63 CAMS; *n* = 244 conventional medicines)

Figure 2 describes the overall concordance and discordance between medications reported by patients and documented in their medical records and the ELMs database. A total of 246 medications (including CAMs) were reported by the cohort of 50 patients and 160 were documented in their medical records. Overall, 125 of 281 medications (44.5 %) were concordant between the patient and their medical record. Twenty-six medications documented in ELMs were not reported by patients or documented in their medical record; these included records of insulin, liver, cardiovascular and respiratory medicines. A large proportion of patient-reported medications that were not documented in the medical record were CAMs.

**Fig. 3:**
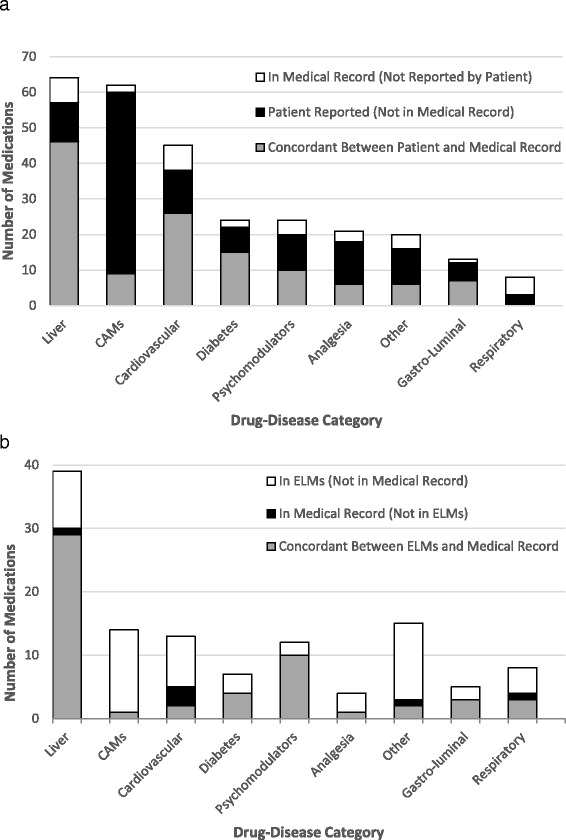
**a**. Concordance between medications reported by the patient and documented in their medical record with respect to drug-disease category. Patients (*n* = 50) taking ≥1 medication in drug-disease class: liver *n* = 28; CAMs *n* = 28; cardiovascular *n* = 22; diabetes *n* = 14; psychomodulators *n* = 13; analgesia *n* = 17; other *n* = 14; gastro-luminal *n* = 10; respiratory *n* = 5. **b**. Concordance between medications recorded in ELMs and documented in the medical record with respect to drug-disease category. Patients (*n* = 16) taking ≥1 medication in drug-disease class: liver *n* = 11; CAMs *n* = 10; cardiovascular *n* = 7; diabetes *n* = 5; psychomodulators *n* = 3; analgesia *n* = 5; other *n* = 5; gastro-luminal *n* = 5; respiratory *n* = 4

The distribution of medication discrepancies by drug-disease class between patients and their medical records, and between the medical record and ELMs is presented in Fig. 3a and 3b respectively. Discrepancies in medications prescribed for the management of liver disease and cirrhosis-related complications are summarised in Table 1. Propranolol and anti-viral therapies were 100 % concordant between the patient and the medical record. Only two of the five patients who were recorded as taking lactulose for hepatic encephalopathy reported using it. The one patient who reported taking trimethoprim-sulfamethoxazole for spontaneous bacterial peritonitis (SBP) prophylaxis did not have this medication documented within their medical record.

**Table 1:** Discrepancies between reported and documented medications prescribed for the management of liver-related complications

Number of liver medications^a^	Patient reported but not documented in medical record	Documented in medical record but not reported by patient	Concordant medications	Proportion (%) discordant records within drug-disease category
Diuretics *n* = 16	1	2	13	18.8 %
Propranolol *n* = 9	0	0	9	0.0 %
Cholecalciferol *n* = 8	4	0	4	50.0 %
PPIs *n* = 7	2	1	4	42.9 %
Thiamine *n* = 6	1	0	5	16.7 %
Lactulose *n* = 5	0	3	2	60.0 %
Antivirals *n* = 4	0	0	4	0.0 %
Other *n* = 9^b^	3	1	5	44.4 %

^a^Excluding 6 liver medications which were only documented in ELMs (*n* = 1 for thiamine, rifaximin, PPI, lactulose, cholecalciferol, spironolactone)

^b^Rifaximin, spontaneous bacterial peritonitis prophylaxis, ursodeoxecholic acid, other vitamins and protein supplements prescribed for complications of cirrhosis

Qualitative analysis of medication discrepancies identified three patients using benzodiazepines and five patients who were taking opiates or non-steroidal anti-inflammatory analgesics not documented in their medical record. One patient took moclobemide, a monoamine-oxidase inhibitor (MAOI) which was not documented, and two patients had angiotensin II receptor antagonists documented in the chart, but not reported by the patient. Five patients had discrepancies involving insulin. Only two of the six patients with documented inhalers reported using them.

### Factors associated with medication discrepancy

The demographic and clinical characteristics of patients according to the presence or absence of medication discrepancies between the patient and medical record is summarised in Table 2. Patients with ≥1 medication discrepancy were older (*p* = 0.04), more likely to be taking ≥5 conventional medications (*p* = 0.01), had a regular GP (*p* = 0.04), comorbidities (*p* = 0.02) and a lower adherence ranking (*p* < 0.01). Multivariate analysis identified the total number of conventional medications and the MMAS ranking as the most significant predictors of discrepancy (Table 3). Patients taking ≥5 conventional medications were 11.0 (95 % CI 1.8–67.4) times more likely to have at least one discrepancy; those with a ‘low’ or ‘medium’ adherence ranking were 20.7 (95 % CI 1.3–337.7) and 49.0 (95 % CI 3.3–718.5) times more likely to have at least one medication discrepancy compared to those with a ‘high’ MMAS ranking.

**Table 2:** Demographic and clinical characteristics for patients with and without medication discrepancies between the patient and their medical record

		≥1 Medication Discrepancy *n* = 27	Medication Discrepancy Absent *n* = 23	*P*
Age, mean (±SD)		61 ± 8	55 ± 11	0.04
Male, no (%)		20 (74.1 %)	19 (82.6 %)	0.52
Years attending clinic, median (range)		2.6 (0.0 – 19.4)	2.9 (0.1 - 13.4)	0.86
Liver disease severity	Compensated Decompensated	55.6 % 44.4 %	65.2 % 34.8 %	0.49
Patient has a regular GP		96.3 %	73.9 %	0.04
Comorbidities present^a^		85.2 %	52.2 %	0.02
Level of Education	Primary/High School	66.7 %	73.9 %	0.58
	Higher Education^b^	33.3 %	26.1 %	
Currently employed		25.9 %	43.5 %	0.19
Patient reported being ‘told how to take your medications’^c^		59.3 %	50.0 %	0.56
Patient reported being able to afford medications^c^		59.3 %	81.2 %	0.19
No. of conventional medicines^c^	1-4 ≥5	22.2 % 77.8 %	62.5 % 37.5 %	<0.01
Adherence ranking (MMAS-8)^d^	High Medium Low	4.2 % 70.8 % 25.0 %	37.5 % 31.2 % 31.2 %	<0.01

^a^Comorbidities included cardiovascular disease, hypertension, diabetes, gastro-oesophageal reflux disease, hypothyroidism, benign prostatic hyperplasia, osteoporosis, rheumatoid arthritis, depression, anxiety, schizophrenia, asthma, chronic obstructive pulmonary disease, and neuropathic pain

^b^Trade, technical certificate, diploma

^c^Excluding 4 patients who took no medications, 2 patients who only took CAMs and 1 patient who did not answer the question (total *n* = 43 patients; ≥1 significant discrepancy *n* = 27; no significant discrepancy *n* = 16). Conventional medicines included vitamins and protein supplements prescribed for the management of cirrhosis or other medical conditions (including: vitamin B1, vitamin D, vitamin A, ferrous sulphate in 1 patient with chronic anaemia, magnesium for 2 patients with symptomatic hypomagnesemia due to diuretic use, and calcium in 1 patient with osteoporosis)

^d^Excluding 10 patients who did not complete this section of the questionnaire (total *n* = 40; ≥1 medication discrepancy *n* = 24; No medication discrepancy *n* = 16)

**Table 3:** Crude and multivariate predictors of medication discrepancies

		Crude OR (95 % CI)	Adjusted OR^a^ (95 % CI)
Age ≥60		1.5 (0.5 – 4.7)	0.9 (0.2 – 4.7)
Male gender		0.6 (0.2 – 2.4)	1.0 (0.1 – 6.6)
Regular GP		9.2 (1.0 – 83.1)	-
≥1 Comorbidity		5.3 (1.4 – 20.1)	2.8 (0.3 – 23.9)
≥5 Conventional Medicines		5.8 (1.5 – 22.7)	11.0 (1.8 – 67.4)
MMAS ranking	Low	7.2 (0.6 – 81.5)	20.7 (1.3 – 337.7)
	Medium	20.4 (2.00 – 211.89)	49.0 (3.3 – 718.5)

^a^Odds ratio adjusted for number of conventional medicines and the MMAS score. Analysis excludes 10 patients who did not complete the MMAS section of the questionnaire

Of the 20 participants who had decompensated cirrhosis, five had a history of hepatic encephalopathy. Encephalopathy was not associated with medication discrepancies in the whole group (*n* = 50, *p* = 0.36), nor in the subset of decompensated patients (*n* = 20, *p* = 0.60) although this finding may be limited by sample size.

### Over-the-counter (OTC), complementary and alternative medications (CAMs)

When initially asked to list their medications, only 31.8 % of over-the-counter, complementary and alternative medications were volunteered by patients. Further specific questioning about OTC products and CAMs were required to elicit these medicines. In total, twenty-seven patients reported taking CAMs, including two patients who stated that they took no medications at all. Only 14.5 % of CAMs reported by all patients were recorded in the medical record, whereas ELMs had a 60.0 % concordance rate within the group of 16 patients who had records in this database.

### Barriers to knowledge and adherence

Of the 43 patients who reported taking medications, only 24 patients (56.0 %) recalled being told how to take them. Eighty-five percent of decompensated patients reported being told to maintain a low salt diet compared to 40 % of compensated cirrhotics (*p* < 0.01), which is consistent with disease management of ascites. Decompensated patients were also more likely to be taking diuretics (*p* < 0.01), but less than one-third knew to keep a record of weight and blood pressure which can both be variably affected by disease and pharmacotherapy.

Fourteen patients (33.0 %) stated that they could not afford their medications, though this was not found to be related to employment status, polypharmacy or disease severity (*p* > 0.05). Of the 40 patients who completed the adherence tool, only 7 were categorised as having ‘high’ adherence.

## Discussion

In this sample of patients with cirrhosis, over half had at least one discrepancy between their reported medicines and those documented in their medical records. Overall concordance between patients and their medical records was under 50 %. Those patients with a discrepancy were more likely to be taking ≥5 medicines and have a medium to low medication adherence ranking.

Discrepancies among CAMs were not unexpected as miscommunication between patients and prescribers on this subject is known to be extensive [14]. However, much like conventional medicines, CAMs are not without potential harm. Adverse reactions are not uncommon [15], and a number of herbal remedies and dietary supplements have been linked to drug-induced liver injury, including traditional Chinese medicines (xiao-chai-hu-tang, rheum palmatum (rhubarb), shou-wu pian), green tea extract, greater celandine, and chaparral [16, 17]. A number of these CAMs are purported to have benefits for patients with pre-existing liver disease, therefore cirrhosis patients who are dissatisfied with conventional medicine may seek out these agents. Hepatologists should be aware of this and actively ask patients about their alternative medication use.

A number of discrepancies among conventional medications had potential for patient harm, such as the misdocumentation or misreporting of insulin, analgesics, benzodiazepines and a MAOI. Errors involving insulin can lead to hospitalisation, MAOIs have potential for severe drug-drug interactions, opioids have reduced clearance in cirrhosis and increased risk of constipation and hepatic encephalopathy, and NSAIDs may contribute to renal impairment and hepatorenal syndrome. Discrepancies involving SBP prophylaxis, diuretics and lactulose among patients with decompensated cirrhosis are also cause for concern, as failure to appropriately manage or monitor these medicines may contribute to hospitalisation with life-threatening decompensation events.

Patients with decompensated cirrhosis average two to three hospital admissions per year [3, 5, 18]. Upon hospitalisation many patients are too unwell to discuss their current medications and may therefore be administered a regimen according to the documented list, which contains discrepancies. Unresolved medication discrepancies have been linked to prolonged hospital stay in people with other chronic diseases [8]. With recurrent hospitalisation, additional pharmacotherapy is often prescribed to manage complications of cirrhosis [3]. With an increase in pharmacotherapy there is a greater chance for patient-clinician miscommunication about medications and thus patients prescribed complex and frequently changing medication regimens are often reported to have poorer adherence [19]. These factors may be further compounded by varying degrees of encephalopathy in people with advanced cirrhosis; only 7 participants in the present study were ranked as having ‘high’ levels of adherence, which is lower than other chronic diseases [12, 20, 21]. Increasing polypharmacy, intentional and unintentional non-adherence, and discrepancies that arise from patient-clinician miscommunication may contribute to re-hospitalisation.

Among decompensated cirrhotics, the number of medications prescribed at discharge has been found to predict hospitalization rate and time to first hospital readmission, independently of the Model for End-stage Liver Disease (MELD) score and serum sodium which also predict poor outcomes [5]. Volk and colleagues estimated that 22 % of 30-day readmissions among patients with decompensated cirrhosis were possibly preventable with improved patient understanding of their medications or more frequent outpatient monitoring [5]. Improved patient understanding may partially be achieved by simplification of the prescribed regimen, which may further improve reporting and adherence due to ease of memory, reduction in side effects and general patient satisfaction [22–24]. However this is difficult to achieve without knowledge of the patient’s entire medication regimen. Routine medication reconciliation within the hepatology clinic may improve this.

In existing outpatient models of collaborative practice, pharmacists have a designated role in medication education and reconciliation, with a number of studies concluding pharmacist intervention reduced hospital admissions, increased adherence to therapy and improved patient outcomes [25–27]. Enhancing the level of disease education in patients of a low educational background has been shown to improve medication adherence [28–31], and use of multiple sources to construct an accurate medication record and identify medication-related problems reduces patient harm [32, 33]. Implementation of a pharmacist within this hepatology practice whose role is to focus on medication reconciliation and management may improve patient outcomes.

### Strengths and limitations

The use of face-to-face interviews conducted by a data collector who had experience with chronic liver disease and was familiar with the patient group allowed for directed qualitative expansion of some patient responses. In addition, a pharmacist conducted the ELMs reviews, assisted with the construction of medication-related questions and discrepancy analysis. Whilst interviewer administration accommodated for potential literacy problems, all patients who completed this survey spoke and read English. Therefore cirrhotic patients requiring an interpreter during the consultation were excluded from assessment. Furthermore, the study relied heavily on patient recall as most patients did not bring their medicines or a list of them to clinic. Some patients with cirrhosis have a carer or family member who assists with managing their medications; this person was not always present at the time of the interview. Decompensated patients may also have had low-grade encephalopathy, affecting medication recall. However these factors reflect the clinic scenario existing in reality, which is what this study aimed to investigate.

## Conclusion

This study demonstrates that there is significant discrepancy between medication sources within the hepatology clinic with potential for harm or impaired disease management. While the aforementioned limitations and single-centre nature of the study may impact on applicability of findings to other sites, we have identified an important potential barrier to care, which may present in similar general hepatology models of care globally. There is much room for improvement in medication reconciliation within the clinic, and our patients may benefit from targeted medication-management intervention.

## Abbreviations

NAFLD: Non-alcoholic fatty liver disease
CLD: Chronic liver disease
CAMs: Complementary and alternative medicines
OTC: Over-the-counter
MMAS: Morisky Medication Adherence Scale
ELMs: Enterprise-wide Liaison Medication System
GP: General practitioner
PPI: Proton pump inhibitor
CI: Confidence interval
OR: Odds ratio
SBP: Spontaneous bacterial peritonitis
MAOI: Monoamine oxidase inhibitor
MELD: Model for End-stage Liver Disease
